# Fracture analysis-based mode-I stress intensity factors of crack under fracture grouting in elastic–plastic soils

**DOI:** 10.1038/s41598-023-28649-2

**Published:** 2023-01-25

**Authors:** Long Li, Yousheng Deng

**Affiliations:** College of Architecture and Civil Engineering, Xi’an University of Science and Technology, Xi’an, 710054 China

**Keywords:** Civil engineering, Applied mathematics

## Abstract

For the interaction of crack on the soil–grout interface under fracture grouting, an approximate method to determine the stress intensity factor (SIF) of crack on the soil–grout interface was proposed based on the conservation *J*_2_-integral. With this method, the energy release rate of crack propagation under fracture grouting can be defined by the parameters of elastic–plastic soils and the grouting pressure. In order to study the change of strain energy near the crack of elastic–plastic soil under fracture grouting, a mechanical model of elastic–plastic soil with crack was established based on non-associated Mohr–Coulomb criterion model, and the SIF of crack with spring boundary was investigated. The influence of the crack depth ratio and crack aspect ratio on the SIF of cracks under the spring boundary were analyzed, and revealed the rule of crack growth under fracture grouting in elastic–plastic soils. The results showed that the variation of the crack depth ratio and crack aspect ratio had an effect on the change of the SIF of cracks. Increasing the crack depth ratio and crack aspect ratio caused an increase in the SIF of the crack. The results can provide the reference for foundation reinforcement in elastic–plastic soils.

## Introduction

Grouting technology is widely used in foundation engineering, which has a good effect in increase the overall stiffness and strength of composite foundation, and improve the bearing capacity of elastic–plastic soils^1^, and it has become an effective technical means to reinforce the elastic–plastic soils by grouting. But the selection of some grouting parameters is judged by subjective experience, and current researches are still farbehind the engineering application^2^. Although many methods, such as Monte Carlo simulation method^3^, Cavity expansion theory^4^, Hoek–Brown et al. strength criterion-based method, have been used to analyze the mechanics of fracture grouting in elastic–plastic soils, it is difficult to comprehensively consider the characteristics of elastic–plastic soils fracture grouting pressure, physical and mechanical properties of elastic–plastic soils and the rule of cracks growth under fracture grouting in elastic–plastic soils from classical method. The research of grouting mechanism is still at the stage of qualitative and elastic mechanics analysis^5^. Therefore, it would be necessary to further study the interaction of the soil–grout interface for crack on the soil–grout interface under fracture grouting.

As an important parameter for evaluating fracture toughness, stress intensity factors plays a significant role in crack growth analysis^6^. Solutions based on two-dimensional theories of fracture are accurate enough for many practical problems. However, Considering the complexity of fracture grouting process, three-dimensional fracture analysis is needed to fully understand fracture behavior of materials, and the study of three-dimensional fracture has been an important issue in fracture mechanics. Theoretical investigations three-dimensional finite boundary crack problems began in late 1980s, Nakamura et al.^7,8^ based on very detailed full-field finite element analysis of the near tip region of a thin isotropic elastic/ductile plate, the three-dimensional stress state in the vicinity of a through-crack front is characterized. More recent analytical solution to depict the relationship among out-of-plane constraints, three-dimensional *J*-integrals and stress intensity factors have been presented by Yi^9,10^ and· Wang^11^, who combined with three-dimensional Maxwell stress functions, the principle of minimum complementary potential energy and three-dimensional *J*-integrals, respectively. Simple solution methods solving the SIFs of elastic–plastic soils under fracture grouting has attracted the attention from many researchers. In 2008, Zou et al.^12^ assumed that the fracture grouting pressure can be determined through the nonlinear Hoek–Brown strength criterion in fracture mechanics and an empirical technique was proposed to determine the SIFs. In 2010, Han et al.^13^ presented a convenient method to estimate grouting pressure during fracture grouting, and claimed that the boundary of fracture grouting and elastic–plastic soils can be expressed as an ellipse, approximately. In 2016, Guo et al.^14^. derived the analytical solution of the equivalent elastic modulus of the two-dimensional model based on the plane model of composite soil in the elastic stage after fracture grouting. Based on fracture mechanics, Wang et al.^15^ proposed a convenient method to estimate the SIFs of elastic–plastic soils under fracture grouting. The theoretical relationship between the grouting pressure and the SIFs of elastic–plastic soils had been derived based on the crack propagation and fracture criterion. It can be known from the research that the cracks propagation is related to the stress intensity factor. When the SIFs of the crack is less than its fracture toughness, the crack will not continue to expand, that is, the crack is in a temporary stable state; when the SIFs of a crack is equal to or greater than its fracture toughness, the crack will continue to propagate. Zhang et al.^16,17^ has verified the effectiveness of Wang’s method in practical engineering and put forward a simple method suitable for engineering application. At the same time, in order to find a simple method to calculate the stress intensity factor, some scholars have done a lot of work, such as Bezuijen et al.^18^, Gu et al.^19^. However, most studies have neglected the plastic deformation of soil, and it was difficult to understand the change of strain energy near the crack of elastic–plastic soil under fracture grouting by stress intensity factor (SIF) of crack on the soil–grout interface.

In present work, some additional simple relationships of the compressive energy density, stress intensity factor and fracture pressure were proposed, which, combined with the *J*_2_-integral, established a calculation method to analyze the SIFs of crack tip under fracture grouting in elastic–plastic soils.

## Theoretical method

### Pressure distribution on the soil–grout interface under fracture grouting

**Figure 1:**
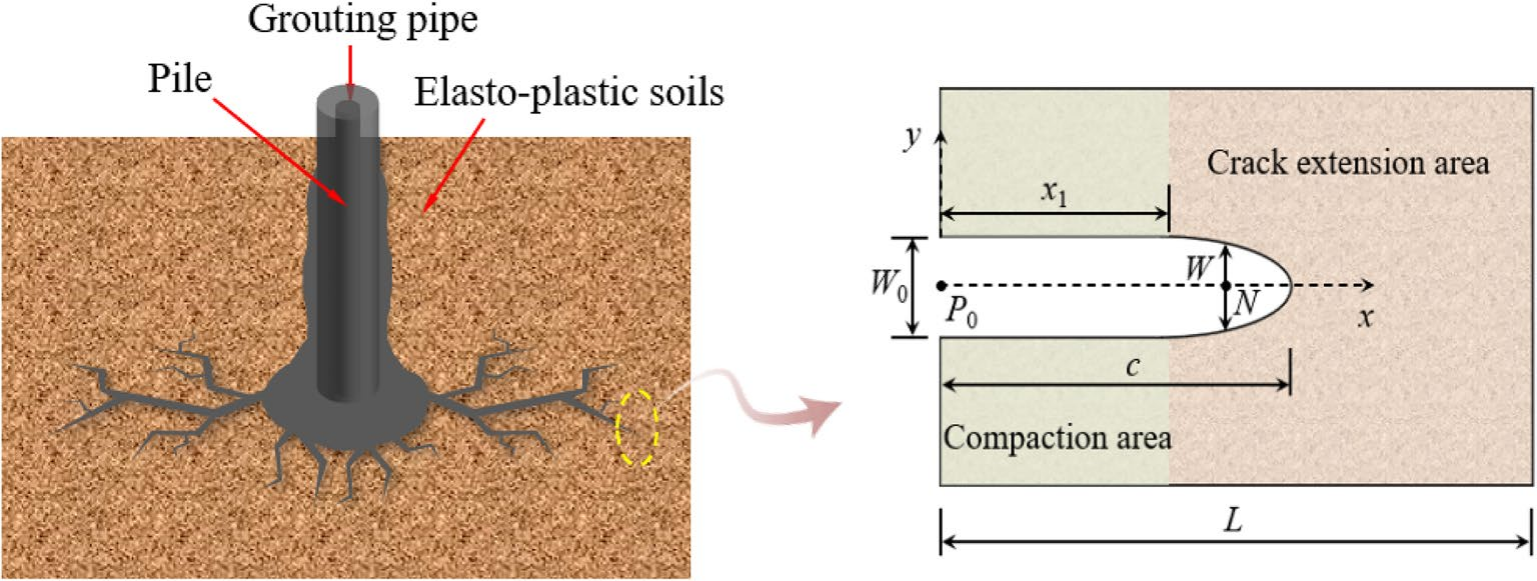
Crack propagation model of fracture grouting.

During the fracture grouting, the pressure exerted on the elastic–plastic soils can be divided into vertical principal stress and horizontal principal stress as shown in Fig. 1. Take the soil–grout interface as the crack surface, then according to two-dimensional flow field theory, the pressure on the crack surface can be calculated by converting the two-dimensional flow on the crack surface into one-dimensional flow^20^.

Schematic diagram of fracture grouting as shown in Fig. 1. *L* represents the parameter of elastic–plastic soil model, *W*_0_ represents the crack width at point *P*_0_ of compaction area, *W* represents the crack width at point *N* of the crack extension area, *x*_1_ represents the crack length of compaction area, *c* represents the total length of crack.

From the reference^21^, a simplified calculation of the pressure on the crack surface during fracture grouting can be expressed as

1 $$ p{ = }\left\{ \begin{gathered} - \frac{{12\zeta qL_{1} }}{{h_{f} w_{0}^{3} \sqrt {L_{{}}^{2} - L_{1}^{2} } }}{ + }P_{0} \, p \ge p_{s} \hfill \\ p_{{\text{s}}} , \, p{ < }p_{{\text{s}}} \, \hfill \\ \end{gathered} \right. $$

where *P*_0_ $\propto$(*c*, *φ*) represents initial pressure when crack propagation, *ζ* represents the viscosity factor of grout, *q* represents the volume flow rate per unit length in *y* direction, *h*_*f*_ represents the height of the crack, and *p*_s_ represents the seepage pressure of elastic–plastic soils.

**Figure 2:**
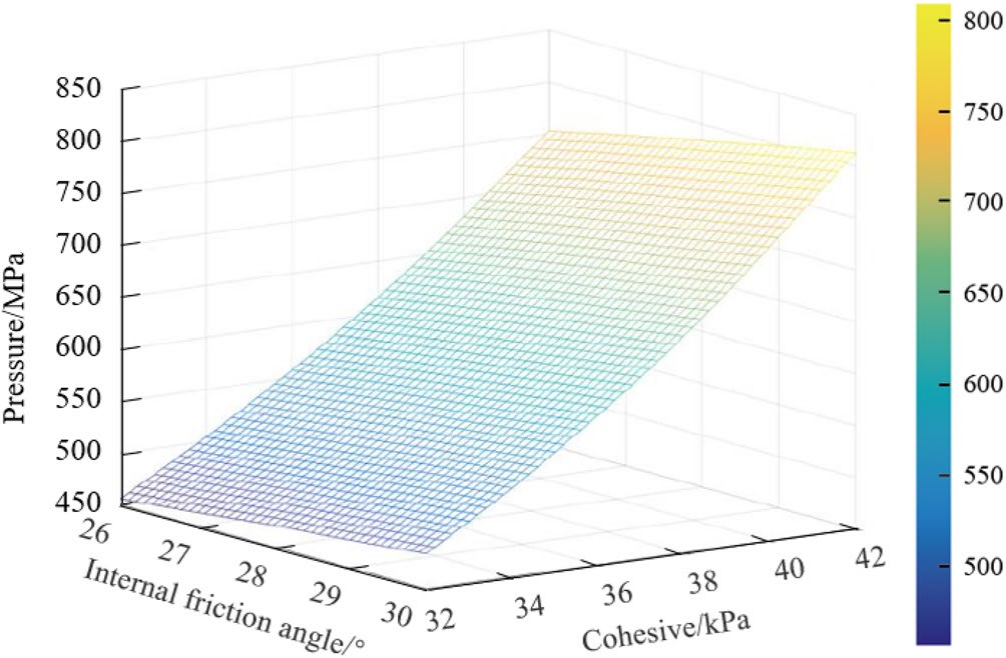
The influence of internal friction angle and cohesion of elastic–plastic soils on fracture pressure.

The variation of fracture pressure with elastic–plastic soils parameters (internal friction angle and cohesion) is shown in Fig. 2.

### J_***2***_ integral

**Figure 3:**
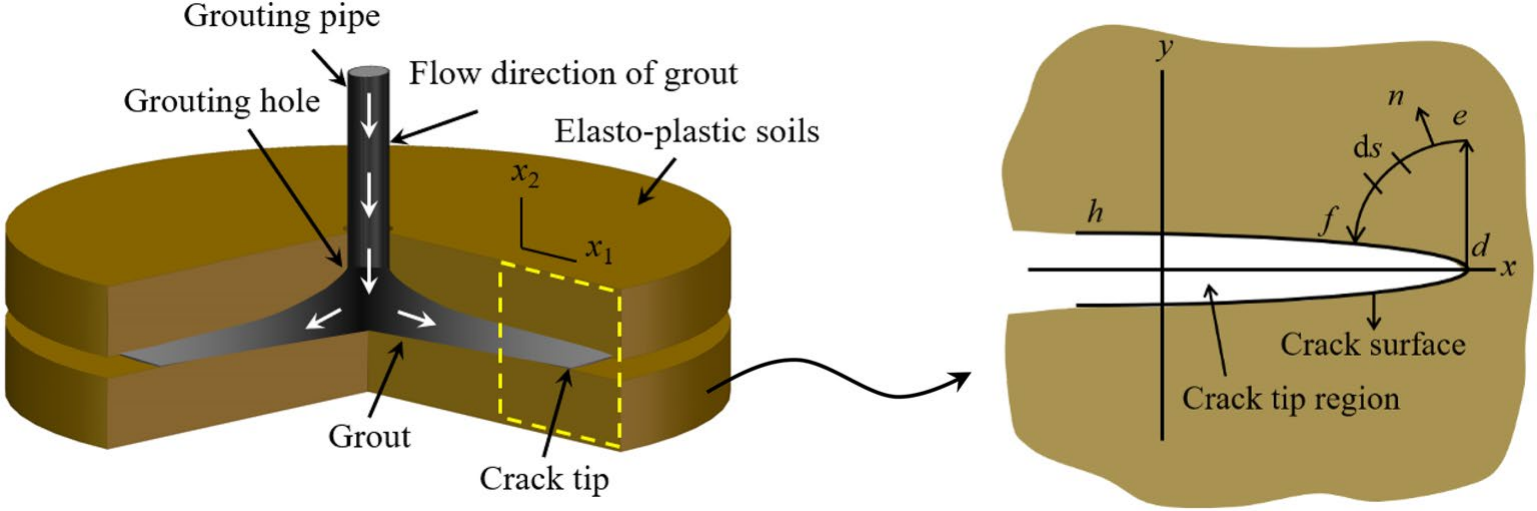
Integration path and K-dominant region.

In order to calculate the stress intensity factor at the crack tip during fracture grouting, the K-dominated integral path at the crack tip is established based on the fracture mechanics theory, as shown in Fig. 3, where *u* represents the displacement vector related to axis *x* and *y*, *S*_defd_ represents a closed path within K-dominant region around grouting expansion surface, *S*_de_ is a straight line and *S*_ef_ is a quarter of circle. For the integral path *S* = *S*_de_ + *S*_ef_—*S*_fd_, from the *J*_2_ integral, we can get^22^

2 $$ J_{2} = \int_{{S_{{{\text{def}}}} }} {\left( {wn_{2} - T_{i} u_{i,2} } \right)} {\text{d}}s = \frac{{\left( {1 - \mu^{2} } \right)K_{{\text{I}}}^{2} }}{{2{\uppi }E}} $$

For the closed path *S*_defd_ = *S*_def_ + *S*_df_, we have

3 $$ \begin{gathered} \int_{{S_{{{\text{defd}}}} }} {\left( {wn_{2} - T_{i} u_{i,2} } \right)} {\text{d}}s \hfill \\ { = }\int_{{S_{{{\text{def}}}} }} {\left( {wn_{2} - T_{i} u_{i,2} } \right)} {\text{d}}s + \int_{{S_{{{\text{df}}}} }} {\left( {wn_{2} - T_{i} u_{i,2} } \right)} {\text{d}}s = \frac{{\left( {1 - \mu^{2} } \right)K_{{\text{I}}}^{2} }}{{2{\uppi }E}} + \int_{{S_{{{\text{df}}}} }} {\left( {wn_{2} - T_{i} u_{i,2} } \right)} {\text{d}}s = 0 \hfill \\ \end{gathered} $$

then

4 $$ J_{2} = \int_{{S_{{{\text{df}}}} }} {\left( {wn_{2} - T_{i} u_{i,2} } \right)} {\text{d}}s = - \frac{{\left( {1 - \mu^{2} } \right)K_{{\text{I}}}^{2} }}{{2{\uppi }E}} $$

Equation (4) means the energy rate released by crack propagation along the *y* direction under the action of grout pressure. *E* and *μ* represent the equivalent elastic modulus and equivalent Poisson's ratio of composite elastic–plastic soils, respectively, which value can be obtained from the reference^14^

5 $$ E = \frac{{\lambda HE_{{\text{g}}} }}{{P_{{\text{s}}} { + }k_{2} P_{{\text{g}}} }} $$

6 $$ \mu { = }\frac{{\lambda \left( {\nu_{{\text{g}}} P_{{\text{s}}} + k_{1} \nu_{{\text{s}}} P_{{\text{g}}} } \right) + m\left( {\nu_{{\text{g}}} P_{{\text{s}}} + k_{2} \nu_{{\text{s}}} P_{{\text{g}}} } \right)}}{{2m\left( {P_{{\text{s}}} + k_{2} P_{{\text{g}}} } \right)}} $$

where

$$ R = \left[ {m\left( {1 + \nu_{{\text{s}}} } \right) + \left( {{{E_{{\text{s}}} } \mathord{\left/ {\vphantom {{E_{{\text{s}}} } {E_{{\text{g}}} }}} \right. \kern-0pt} {E_{{\text{g}}} }}} \right)\left( {1 - m} \right)\left( {1 + \nu_{{\text{g}}} } \right)} \right],\;H = P_{{\text{s}}} + k_{1} k_{2} P_{{\text{g}}} + k_{1} P_{{{\text{sg}}}} + k_{2} P_{{{\text{sg}}}} ,\;P_{{\text{s}}} = \left( {1 - \nu_{{\text{s}}} - 2\nu_{{\text{s}}}^{2} } \right),\;P_{{\text{g}}} = \left( {1 - \nu_{{\text{g}}} - 2\nu_{{\text{g}}}^{2} } \right),\;P_{{{\text{sg}}}} = \left( {1 - \nu_{{{\text{sg}}}} - 2\nu_{{\text{s}}} \nu_{{\text{g}}} } \right),\;P_{{{\text{gs}}}} = \left( {1 - \nu_{{{\text{gs}}}} - 2\nu_{{\text{g}}} \nu_{{\text{s}}} } \right),\;k_{1} = \left( {E_{{\text{s}}} /E_{{\text{g}}} } \right){{\left( {1 - \lambda } \right)} \mathord{\left/ {\vphantom {{\left( {1 - \lambda } \right)} \lambda }} \right. \kern-0pt} \lambda } $$

$$ k_{2} = \left( {E_{{\text{s}}} /E_{{\text{g}}} } \right){{\left( {1 - m} \right)} \mathord{\left/ {\vphantom {{\left( {1 - m} \right)} m}} \right. \kern-0pt} m} $$

**Figure 4:**
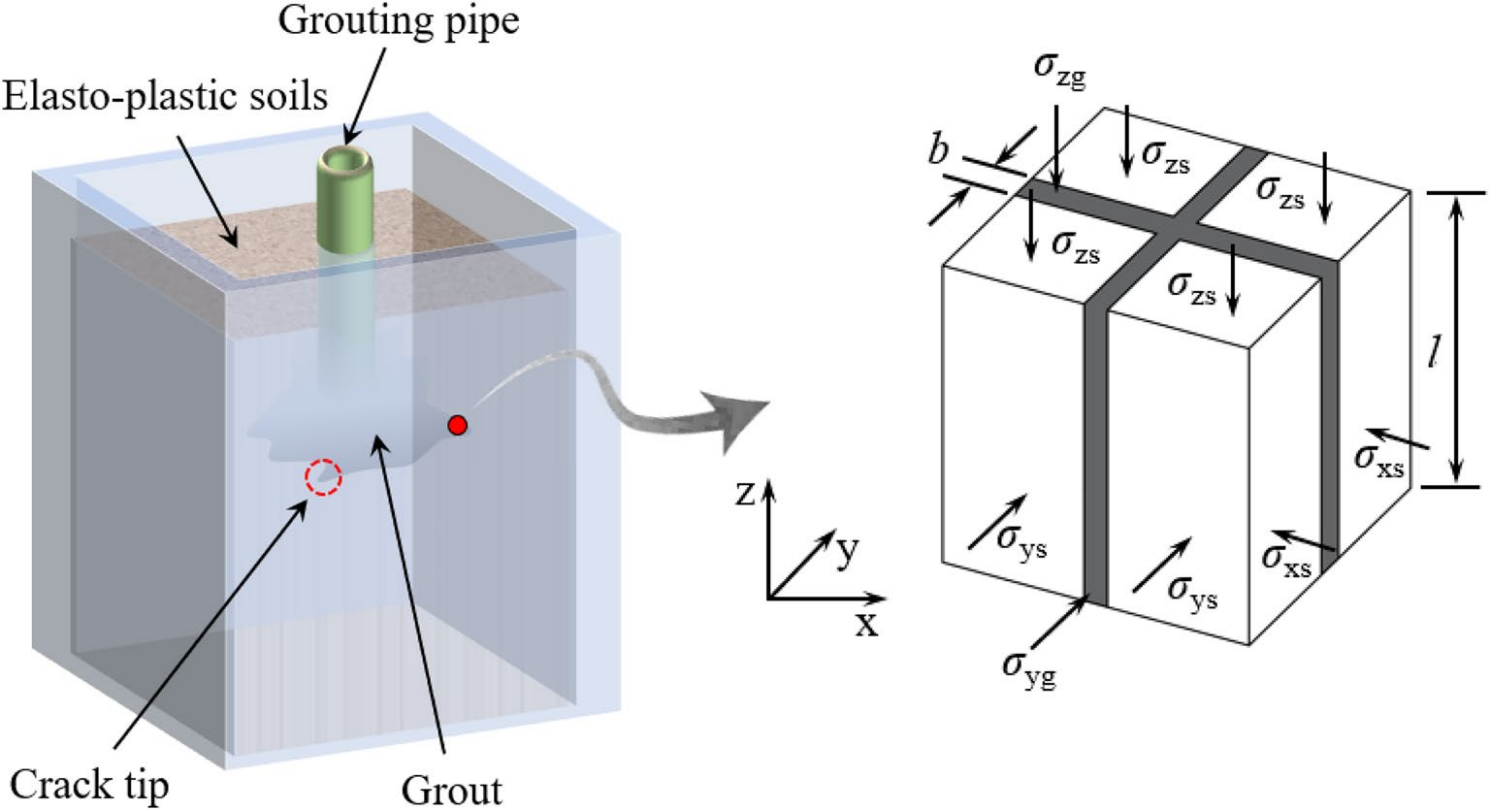
Conceptual model of theoretical procedure.

*E*_s_, *E*_g_, *ν*_s_, *ν*_g_ are the elastic modulus and Poisson's ratio of elastic–plastic soils and grout, respectively. After the elastic–plastic soils been fracture grouted, the composite elastic–plastic soils is equivalent to the transverse isotropic model in macroscopic mechanical properties, and its equivalent unit in rectangular coordinate system is shown in Fig. 4. *σ*_x_, *σ*_y_ and *σ*_z_ represent the normal stress in the corresponding coordinate direction, respectively. The subscripts s and g represent elastic–plastic soils and grout, respectively. *l* represents the side length of the equilateral unit, and $\tilde{w}$ represents the equivalent width of the crack, *m* represents the side equivalent injection rate of grout around crack tip, *λ* represents the actual injection rate of grout, and *m* and *λ*⋅can be expressed as follows.

$$\lambda = \frac{{2\tilde{w}l - \tilde{w}^{2} }}{{l^{2} }}$$, $$m = 1 - \sqrt {1 - \lambda }$$.

### SIFs of crack tip under fracture grouting

**Figure 5:**
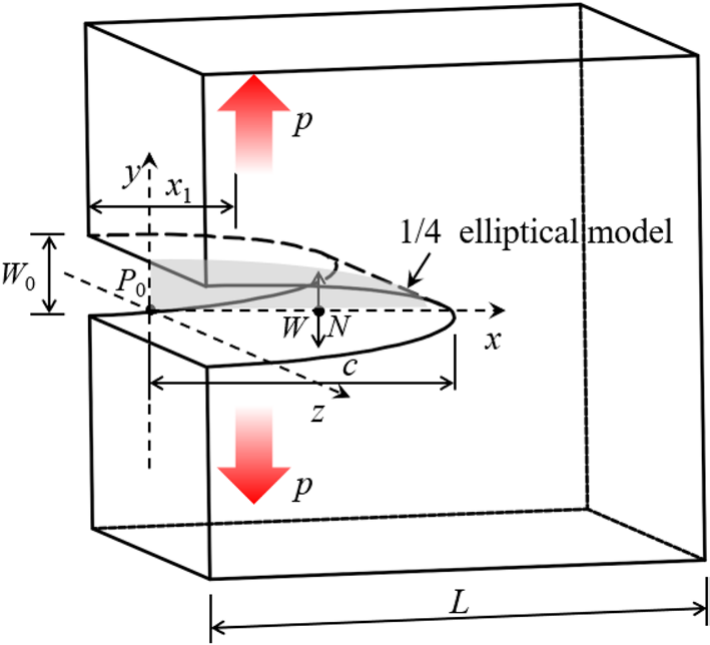
1/4 elliptical modelling for cracks with *W*/2 → 0.

**Figure 6:**
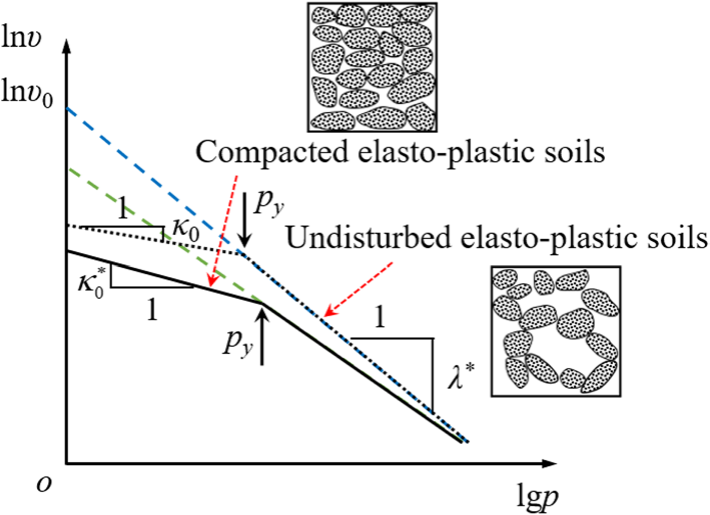
Compression curves of undisturbed and compacted remolded elastic–plastic soils with the same dry density.

When elastic–plastic soils is subjected to fracture grouting, according to the crack propagation law of fracture mechanics^23^, the crack tip of elastic–plastic soils can be simplified as Fig. 5, where *p* represents the grouting pressure on the crack surface. Under the condition of *W*/2 → 0, as for undisturbed elastic–plastic soils, the compressive energy density of elastic–plastic soils in *y* direction can be expressed as

7 $$ \tilde{u}_{y}^{ - } = \frac{{\left( {\lambda^{*} - \kappa_{0}^{*} } \right)\lg^{2} p_{y} + \lg^{2} p\left( {1 + \lambda^{*} } \right)}}{{2\ln \upsilon_{0} }} $$

where λ^*^ is the slope for elastic deformation stage of undisturbed elastic–plastic soils, $\kappa_{0}^{*}$ is the slope for plastic deformation stage of compacted elastic–plastic soils as shown in Fig. 6, *p*_*y*_ is the yield stress of elastic–plastic soils, ln*υ*_0_ is the intercept of the plastic section of undisturbed elastic–plastic soils on the lnυ axis.

The crack can be regard as the degeneration of the elliptical hole when *W*/2 → 0, so the compressive energy density at the crack surface of compacted elastic–plastic soils can be obtained from the following limit

8 $$ \tilde{u}_{y}^{ + } = \mathop {\lim }\limits_{{{W \mathord{\left/ {\vphantom {W 2}} \right. \kern-0pt} 2} \to 0}} \frac{2}{W}\int_{0}^{\frac{W}{2}} {\frac{{p{\text{d}}x}}{{EA\left[ {1 - \left( {{W \mathord{\left/ {\vphantom {W L}} \right. \kern-0pt} L}} \right)/\sqrt {1 - \left( {{{2x} \mathord{\left/ {\vphantom {{2x} W}} \right. \kern-0pt} W}} \right)^{2} } } \right]}}} $$

When the *J*_2_-integral is used to calculate the SIFs of the elastic–plastic soils, at the crack surface of elastic–plastic soils during crack propagation, we can get the SIFs of the elastic–plastic soils by using the 1/4 elliptical model as follows

9 $$ \frac{{\left( {1{ - }\mu^{2} } \right)K_{{\text{I}}}^{2} }}{{{\uppi }E}} + \int_{{S_{{{\text{df}}}} }}^{{}} {w{\text{d}}S = } \frac{p}{2}\left( {\tilde{u}_{y}^{ + } - \tilde{u}_{y}^{ - } } \right) $$

Hence, it is not difficult to get

10 $$ K_{{\text{I}}} { = }\sqrt {\frac{{{\uppi }E}}{{1 - \mu^{2} }}\left[ {\frac{p}{2}\left( {\tilde{u}_{y}^{ + } - \tilde{u}_{y}^{ - } } \right) - \int_{{S_{{{\text{df}}}} }}^{{}} {w{\text{d}}s} } \right]} $$

## Finite element model

### Soil elastic-perfectly plastic (EPP) model and boundary conditions

**Figure 7:**
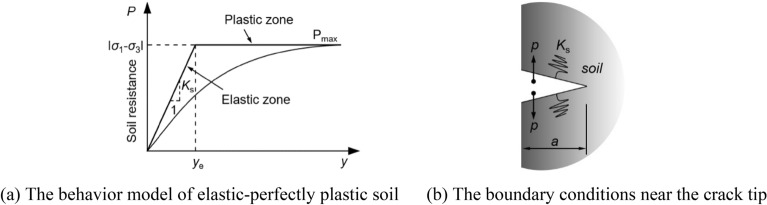
The model configuration of elastic-perfectly plastic soil.

According to the non-associated Mohr–Coulomb criterion model, as shown in Fig. 7a, the mechanical properties of elastic–plastic soil can be expressed as

11 $$ P_{\max } = 2c \cdot \cos \varphi - \left( {\sigma_{1} + \sigma_{3} } \right)\sin \varphi $$

12 $$ y_{{\text{e}}} = P_{\max } /K_{{\text{s}}} $$

13 $$ K_{{\text{s}}} = \frac{{13E_{{\text{s}}} }}{{20L\left( {1 - \nu_{{\text{s}}}^{2} } \right)}}\sqrt {\frac{{E_{{\text{s}}} L^{4} }}{{E_{{\text{b}}} I_{{\text{b}}} }}} $$

where *P*_max_ represents the maximum resistance of elastic–plastic soil, *y*_e_ represents the yield deformation, *K*_s_ is reaction modulus of elastic–plastic soil, *c* is the cohesion of the soil, *φ* is the friction angle the soil, and *E*_b_ is the elastic modulus of soil, *I*_b_ and *L* are, respectively, the inertia and the parameter of elastic–plastic soil model.

In this paper, we use spring boundary to reflect the soil–grout interaction, and the boundary conditions near the crack tip and behavior model of elastic-perfectly plastic soil as shown in Fig. 7b.

### Finite element analysis

**Figure 8:**
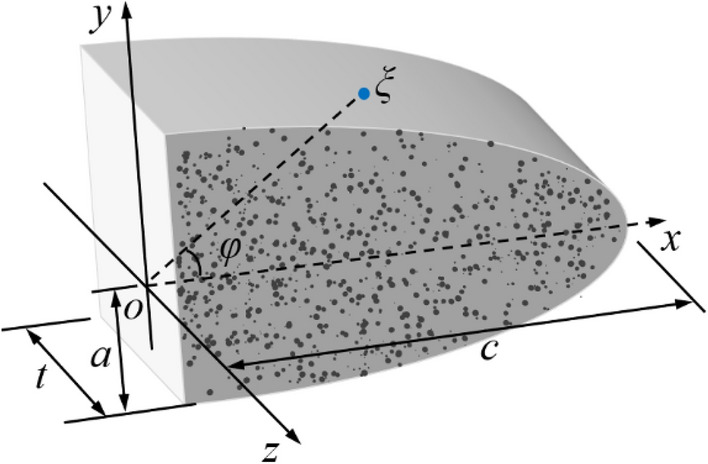
The geometry of 1/4-elliptical crack.

**Figure 9:**
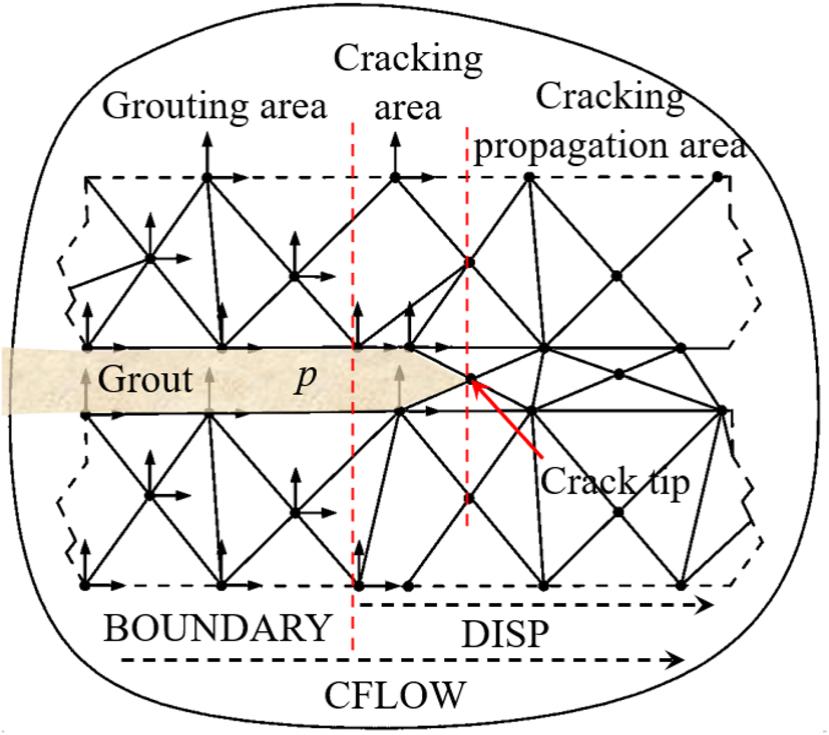
Schematic diagram of finite element analysis model of crack growth.

**Figure 10:**
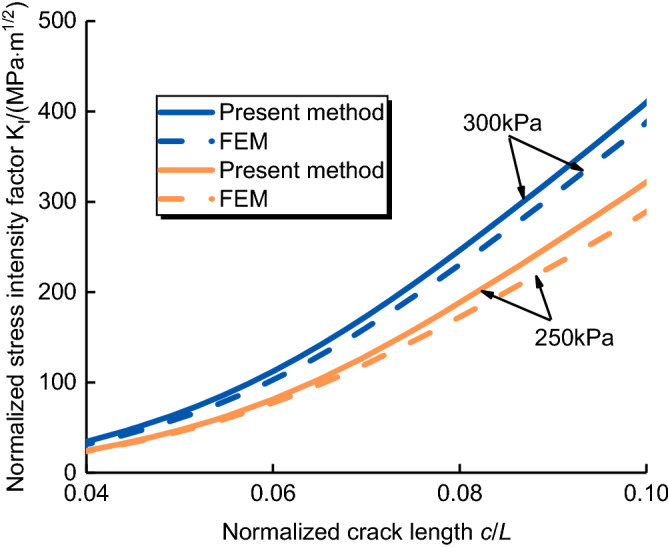
SIFs for crack tip of the elastic–plastic soils under fracture grouting.

In actual engineering, it is difficult to get the crack propagation length of fracture grouting. Most problems should be solved using numerical simulation, so large-scale finite element analysis software-Abaqus has been used in the paper^24^. During the finite element calculation, the crack tip is divided into the crack propagation area and compaction area as shown in Fig. 1. In crack propagation area, the geometry of 1/4-elliptical crack as shown in Fig. 8 can be described by the dimension parameters, *a*, *c*, *t* and *φ*, where *a* is half of the crack mouth displacement, *c* is the distance from the crack mouth to the crack tip, *t* is the thickness of the crack surface in the z-axis direction, *φ* is the angle between the straight line *oξ* and the *x* axis, and *ξ* is any point of crack surface. The angle of crack was 0° and 90° means that the crack does not extended or generate, the crack depth ratio *a*/*t* and the crack aspect ratio *a*/*c* is constant at 0, while the angle of crack was between 0° and 90° means that the crack growth. In numerical simulation, the pressure *p* of the crack surface be inputted by the subroutine CFLOW, and then transmitted to the subroutine DISP in real time according to the BOUNDARY direction as shown in Fig. 9. By analyzing the coordinate values of each node in the grouting area and crack propagation area, the relative distance of each pair of nodes in the crack surface can be obtained, through which can further get the width *W* of the crack propagation area, then the stress intensity factor at the crack tip of elastic–plastic soils can be obtained by combining the traditional xfem crack analysis method as shown in Fig. 10.

The parameters are selected as follow: the fracture toughness of the elastic–plastic soils is 2.7 MN/m^3/2^, the fracture energy in the shear mode is 54.7 J, the cohesive unit selection is COH2D4, and undisturbed elastic–plastic soils mechanics performances are shown in Table 1. Figure 10 shows the SIFs for crack tip of the elastic–plastic soils under fracture grouting of present method (Eq. 10) and FEM.

**Table 1:** Mechanical characteristics of elastic–plastic soils.

Cohesion, *c *(kPa)	Internal fraction angle, *φ *(%)	Water content, *w *(%)	Density, *ρ *(g cm^−3^)
31.2	25.18	17.53	1.73

The SIF of crack tip for elastic–plastic soils is shown in Fig. 10. It is clearly found that the difference between the present method and FEM is very small, under the same conditions, the normalized SIFs increased with the increase of normalized crack length, which trend is similar to the reference^25^. So the present method can reflect the trend of the mechanical characteristics of crack tip for elastic–plastic soils under fracture grouting. Under the grouting pressure of 250 kPa, the SIF at the crack tip of elastic–plastic soil increases with the increase of normalized crack length, and its increase rate is relatively slow within the normalized crack length range of 0.04–0.06, when the normalized crack length approaches 0.1, the normalized stress intensity factor increases rapidly, and the maximum value of SIFs appeared at the range of 285–308 MPa m^1/2^. Under the grouting pressure of 300 kPa, the increasing trend of SIFs at the crack tip of elastic–plastic soil are similar to that under the grouting pressure of 250 kPa, and the maximum value of SIFs appeared at the range of 387–403 MPa m^1/2^. It can be seen that the SIFs of the present method is greater than FEM, which means that the analytical results obtained after homogenization treatment in FEM are reasonable and correct, but conservative. The present method in this paper considers the plastic deformation of elastic–plastic soil and can better describe the mechanical characteristics of soil–grout interaction.

### Example calculation

**Figure 11:**
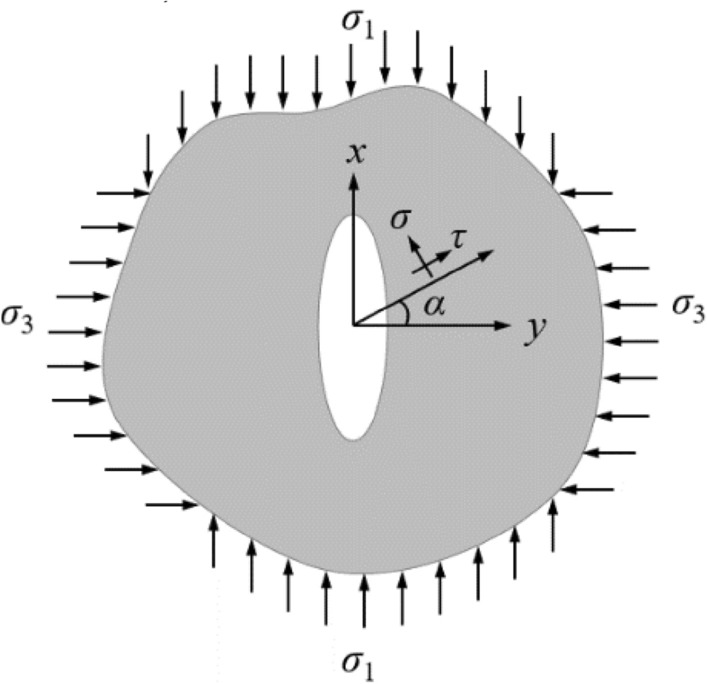
Crack analysis model based on fracture criterion of crack propagation.

In order to verify the accuracy of present method, we calculated the SIF of homogeneous fracture grouting pressure (*p* = 250 kPa) along the elliptic crack front. The crack depth ratio *a*/*t* of 1/4-elliptical surface is 0.3, crack aspect ratio *a*/*c* is 0.3, the crack analysis model based on fracture criterion of crack extension is shown in Fig. 11. the stress near the crack tip of the elastic–plastic soils under the fracture pressure can be expressed as

14 $$ p_{1} = \frac{{\sigma_{1} + \sigma_{3} }}{2} + \frac{{\sigma_{1} - \sigma_{3} }}{2}\left( {\cos 2\sigma - \frac{\sin 2\alpha }{{\tan \alpha }}} \right) $$

When elastic–plastic soils conforms to the rule of fracture mechanics of the propagation of the crack, the SIF of crack tip is equal to the critical SIF of the soil^15^. Fracture criterion of crack propagation is then as follows

15 $$ K_{{\text{I}}} = K_{{{\text{IC}}}} $$

where *K*_I_ denotes the SIF of crack, while *K*_IC_ denotes the fracture toughness. Among them:

16 $$ K_{{\text{I}}} = \frac{p}{E\left( k \right)}\sqrt {\frac{{c{\uppi }}}{a}} (a^{2} \sin^{2} \alpha + c^{2} \cos^{2} \alpha )^{1/4} $$

The parameter *E*(*k*) is the elliptic integrals, and *E*(*k*) can be expressed as

17 $$ E\left( k \right) = \int_{0}^{{{\uppi }/2}} {\left( {1 - \frac{{a^{2} - c^{2} }}{{a^{2} }}\sin^{2} \alpha } \right)}^{{{1 \mathord{\left/ {\vphantom {1 2}} \right. \kern-0pt} 2}}} {\text{d}}\alpha $$

Compared with the literature solution^25^, the Eq. (10) result is very close to the result calculated by Eq. (16), as shown in Table 2.

**Table 2:** Error value between present method and literature solution.

Method	SIFs of crack tip under fracture grouting in elastic–plastic soils (Pa m^1/2^)
Equation (10) result	1,460,450
Equatiom (16) result	1,387,140
The error	5.28%

### Results and discussion

#### Process of fracture grouting

**Figure 12:**
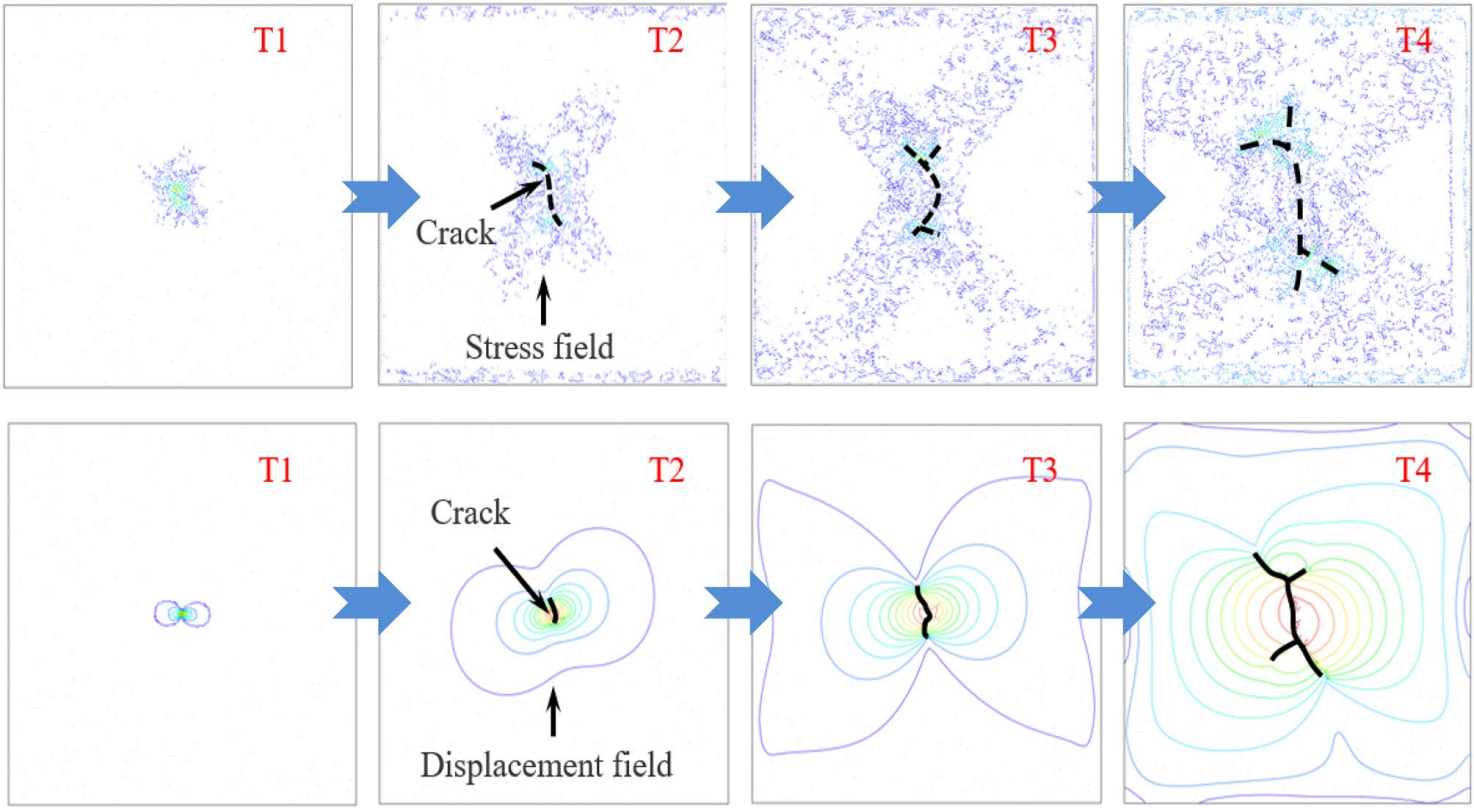
Stress field and displacement field of elastic–plastic soils at different times during fracture grouting.

The process of fracture grouting can be divided into three stages: expansion and compaction stage, fracturing stage and final compaction stage, and the simulation of grouting are shown in Fig. 12. 0 ~ T1 is the stage of soil expansion and compaction. With the continuous increase of the grouting pressure, Within the stage of T1–T2 and T2–T3, the crack spread away from near the grouting hole, at the stage of T3–T4, the grout continuously splits the soil under the pressure of fracture grouting, and the fracture crack of the soil presents an "X" shape. After T4 point, the soil around the grouting hole is compacted, and finally the polymer stone body is formed. In the process of fracture grouting, the variation of stress field and strain field of soil are related to the crack propagation law. To sum up, fracture grouting can strengthen the soil by changing the stress field and strain field, which is a good method for foundation treatment.

#### Effects of crack depth ratio on the SIF of cracks

**Figure 13:**
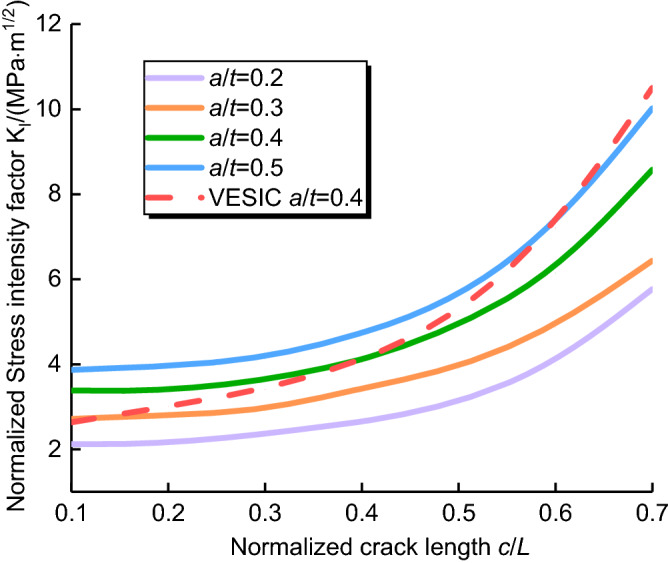
Relationships the normalized stress intensity factor and normalized crack length (0.2 ≤  *a*/*t* ≤ 0.5).

Figure 13 shows the relationship between the normalized stress intensity factor and normalized crack length with the crack aspect ratio 0.2 ≤ *a*/*t* ≤ 0.5. In the case that *a*/*t* = 0.2, 0.3, 0.4 and 0.5, the normalized stress intensity factor increases with the increase of normalized crack length. At the same time, with the increase of *a*/*t*, the normalized SIFs increases continuously, and peaked in *c*/*L* = 0.7, which may result from the increases of energy release of the cracks tip.

The finite element results are compared with the VESIC's method, the FEM results are very close to the semi-empirical relationships^26^ proposed by Vesic as shown in Fig. 13, and the correlativity coefficients *R*^2^ are more than 0.8826.

#### Effects of crack aspect ratio on the SIF of cracks

**Figure 14:**
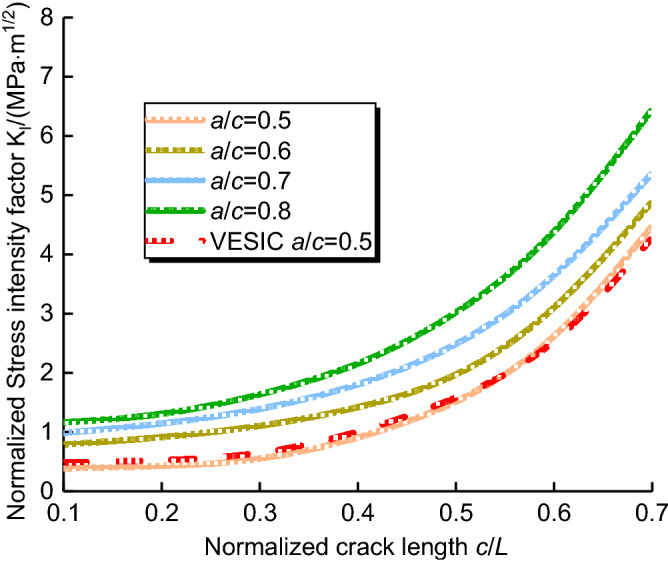
Relationships the normalized stress intensity factor and normalized crack length (0.5 ≤  *a*/*c* ≤ 0.8).

Figure 14 shows the relationship between the normalized stress intensity factor and normalized crack length with the crack aspect ratio 0.5 ≤ *a*/*c* ≤ 0.8. when *a*/*t* = 0.2, 0.3, 0.4 and 0.5, the normalized stress intensity factor increases with the increase of normalized crack length, and the normalized stress intensity factor increases with the increase of crack aspect ratio. When the aspect ratio *a*/*c* = 0.5, the results of finite element results and the VESIC's is greatly consistent, and the correlativity coefficients *R*^2^ are more than 0.9831.

#### Influencing factors of crack propagation

- The grouting pressure

**Figure 15:**
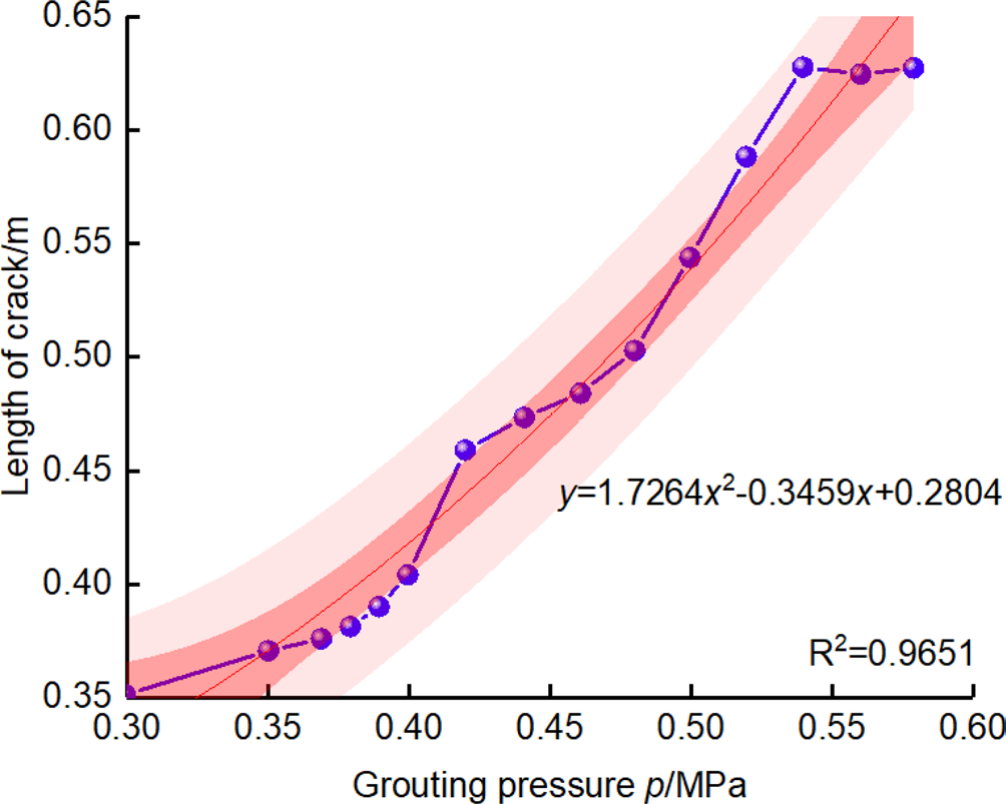
The relationship of crack length and grouting pressure.

In the process of fracture grouting, the pressure of grout promotes the expansion of cracks, changes the original structure of soil, and eliminates the air gap of soil by filling grout, thus achieving the purpose of reinforcement. The relationship between grouting pressure and crack propagation distance is as shown in Fig. 15. It can be seen that the crack length is significantly affected by grouting pressure, with the increase of grouting pressure, the crack length shows a non-linear growth trend, this is because with the increase of grouting pressure, the energy of grout in soil gathers together, one part of the energy is used to overcome the shear stress of soil, and the other part of the energy is used for crack expansion. In addition, compared with the confidence interval given in the literature, the width of the confidence interval of this FEM results is narrower (the pink area as shown in Fig. 15), hence the overestimation can be avoided, indicating the proposed methods of significance in both the current engineering design and the prospective application.

- The grouting time

**Figure 16:**
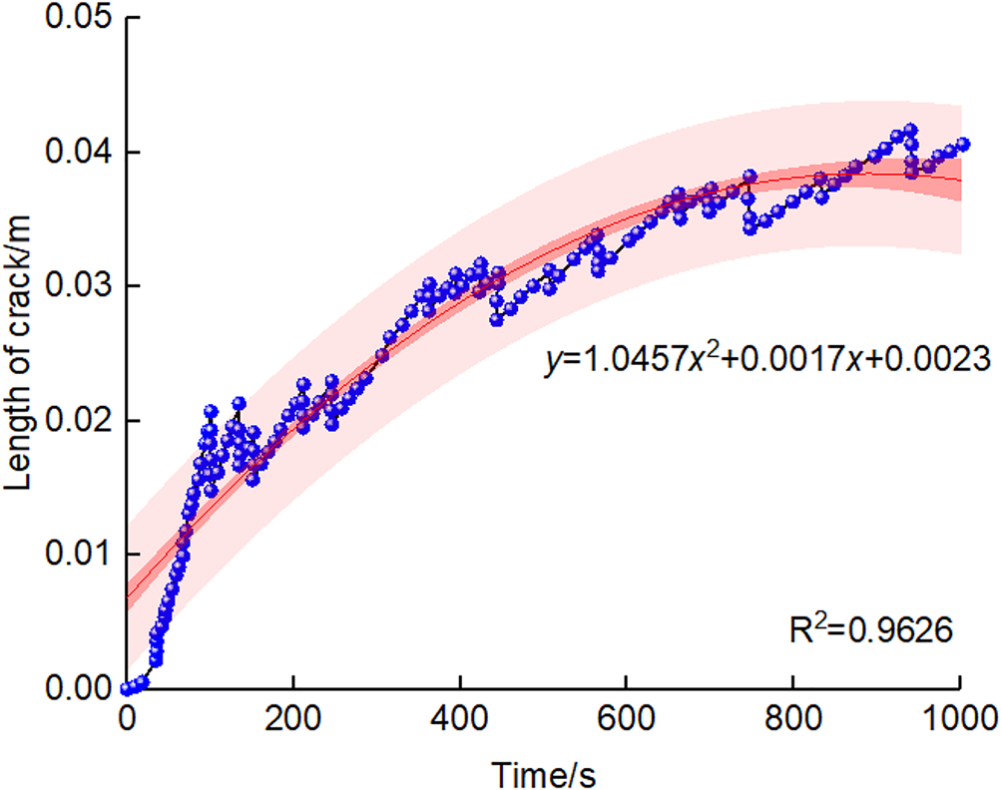
The relationship of crack length and grouting time.

A curve of length of crack changing with the variation of grouting time is shown in Fig. 16, It can be seen that the crack length is significantly affected by grouting time, with the increase of grouting time, the crack length shows a non-linear growth trend, which means that under the same grouting pressure, with the increase of grouting time, the more obvious the fracture effect of grout on soil and the longer the crack length. According to the research of references^27^, the confidence interval of the influence of grouting time on crack length is shown in the pink area in Fig. 16, and the correlativity coefficients *R*^2^ are more than 0.9626.

## Conclusions

Based on the theory of fracture mechanics and finite element theory, this paper analyzes the mechanical characteristics of crack tip in the process of fracture grouting in elastic–plastic soils. Combined on soil elastic-perfectly plastic model and the spring boundary condition, the fracture grouting process of elastic–plastic soils is simulated by theoretical analysis and numerical calculation, and the analytical solution of stress intensity factor at crack tip of elastic–plastic soils in the process of fracture grouting is derived based on *J*_2_ integral, and the influence of crack size on normalized stress intensity factors is analyzed, such as crack depth ratios and crack aspect ratio. The present method describes the generation form for cracks of elastic–plastic soils under fracture grouting, the establishment of method of grouting pressure, normalized crack length and normalized stress intensity factors in the process of fracture grouting in elastic–plastic soil, and realizes the solution of crack propagation problem and energy release under grouting pressure problem. Below are the main conclusions:

1. Based on the fracture mechanics, the process of fracture grouting of elastic–plastic soils under fracture grouting were studied and crack propagation model in elastic–plastic soils was established.
2. Based on the conservation *J*_2_-integral, considering the energy change of elastic–plastic soils crack propagation in the process of fracture grouting, by introducing the equivalent elastic modulus and equivalent Poisson's ratio of elastic–plastic soils under fracture grouting, an analytical solution of stress intensity factor at the crack tip of elastic–plastic soils is derived.
3. The normalized SIF increases with the increase of normalized crack length, the rate of increase for the normalized SIF were gradually increasing with the increase of *a*/*c*, when *a*/*c* = 0.5, 0.6, 0.7 and 0.8, with the increase of *a*/*c*, the normalized SIFs increases continuously, and peaked in *c*/*L* = 0.7. When *a*/*t* = 0.2, 0.3, 0.4 and 0.5, the maximum value of normalized SIFs appears at the maximum of *c*/*L* and the maximum of *a*/*t*, which may result from the increases of energy release of the cracks tip.
4. The incorporation of stress intensity factor in the study of soil–grout interaction and combine with 1/4-elliptical crack model for mechanical characterization of crack, and provides an analysis method for the grouting problem of elastic–plastic soil.
5. The medium studied in this paper is elastic–plastic soil, and the FEM results of cracks in soil under fracture grouting pressure are basically consistent with the existing literature, which verifies that the crack propagation of fracture grouting conforms to the crack propagation theory of fracture mechanics. In the process of field test, the effect of grouting reinforcement can be judged by the configuration of crack tip, which provides theoretical guidance for field reinforcement design.

## Data Availability

Correspondence and requests for materials should be addressed to Long Li.
